# Impact of malaria interventions on child mortality in endemic African settings: comparison and alignment between LiST and Spectrum-Malaria model

**DOI:** 10.1186/s12889-017-4739-0

**Published:** 2017-11-07

**Authors:** Eline Korenromp, Matthew Hamilton, Rachel Sanders, Guy Mahiané, Olivier J. T. Briët, Thomas Smith, William Winfrey, Neff Walker, John Stover

**Affiliations:** 1 Avenir Health, PO box 2100, CH-1211 Geneva, Switzerland; 2 Avenir Health, 655 Winding Brook Drive, Glastonbury, CT-06033 USA; 3 Epidemiology and Public Health, Swiss Tropical and Public Health Institute, Socinstrasse 57, 4051 Basel, Switzerland; 4 Epidemiology and Public Health, University of Basel, Basel, Switzerland; 5 Department of International Health, Institute for International Programs, Johns Hopkins University Bloomberg School of Public Health, 615 N. Wolfe St., Baltimore, MD 21205 USA

**Keywords:** Malaria, Child health, Model, Prevention, Treatment, Health impact, Mortality, Africa

## Abstract

**Background:**

In malaria-endemic countries, malaria prevention and treatment are critical for child health. In the context of intervention scale-up and rapid changes in endemicity, projections of intervention impact and optimized program scale-up strategies need to take into account the consequent dynamics of transmission and immunity.

**Methods:**

The new Spectrum-Malaria program planning tool was used to project health impacts of Insecticide-Treated mosquito Nets (ITNs) and effective management of uncomplicated malaria cases (CMU), among other interventions, on malaria infection prevalence, case incidence and mortality in children 0–4 years, 5–14 years of age and adults. Spectrum-Malaria uses statistical models fitted to simulations of the dynamic effects of increasing intervention coverage on these burdens as a function of baseline malaria endemicity, seasonality in transmission and malaria intervention coverage levels (estimated for years 2000 to 2015 by the World Health Organization and Malaria Atlas Project). Spectrum-Malaria projections of proportional reductions in under-five malaria mortality were compared with those of the Lives Saved Tool (LiST) for the Democratic Republic of the Congo and Zambia, for given (standardized) scenarios of ITN and/or CMU scale-up over 2016–2030.

**Results:**

Proportional mortality reductions over the first two years following scale-up of ITNs from near-zero baselines to moderately higher coverages align well between LiST and Spectrum-Malaria —as expected since both models were fitted to cluster-randomized ITN trials in moderate-to-high-endemic settings with 2-year durations. For further scale-up from moderately high ITN coverage to near-universal coverage (as currently relevant for strategic planning for many countries), Spectrum-Malaria predicts smaller additional ITN impacts than LiST, reflecting progressive saturation. For CMU, especially in the longer term (over 2022–2030) and for lower-endemic settings (like Zambia), Spectrum-Malaria projects larger proportional impacts, reflecting onward dynamic effects not fully captured by LiST.

**Conclusions:**

Spectrum-Malaria complements LiST by extending the scope of malaria interventions, program packages and health outcomes that can be evaluated for policy making and strategic planning within and beyond the perspective of child survival.

**Electronic supplementary material:**

The online version of this article (10.1186/s12889-017-4739-0) contains supplementary material, which is available to authorized users.

## Background

In malaria-endemic countries, malaria prevention and treatment are critical for child health. Between 2000 and 2015, malaria incidence rates fell 37% globally, and malaria mortality rates by 60%, with even greater declines in Africa, the highest-burden region [1]. This was likely a combined result of improved malaria control and other factors independent of interventions [2]. Across all age groups in sub-Saharan Africa, malaria control interventions accounted for an estimated 70% of the 943 million fewer malaria cases occurring between 2001 and 2015. For malaria deaths in children under-5, in 2012 the World Health Organization (WHO) had estimated that malaria prevention intervention scale-up over 2001–2010 had prevented 921,300 (uncertainty interval: 625,600–1,260,800) child deaths (or 8.2% of the number in 2000) due to malaria across 36 malaria-endemic countries in Africa [3]. Impacts of improved case management, as well as mortality and morbidity impacts in older children and adults, are less certain.

As funding for malaria control has now plateaued, it is even more critical than before to prioritize interventions with the most impact, and determine and plan the optimal mix of interventions for each setting and time period —within the broader public health and health sector context. We developed a malaria strategic planning module in the Spectrum suite of policy models, to complement similar modules included for HIV/AIDS, tuberculosis and family planning, which are used by over 120 low and middle-income countries for estimation of burdens, trends, service needs and program impact [4–7]. The Spectrum-Malaria module extends the malaria impact modelling options available through the Lives Saved Tool (LiST) [3, 8, 9], by projecting impacts on not only mortality but also morbidity (malaria case incidence and prevalence of infection with *Plasmodium falciparum* (*Pf*PR), for not only children 0–4 years but also children 5–14 years and adults. Besides Insecticide-Treated mosquito Nets (ITNs), Indoor Residual Spraying (IRS) and first-line treatment of malaria cases, also modelled in LiST, Spectrum-Malaria additionally projects the impact of severe case management of sever malaria cases, and seasonal malaria chemoprophylaxis for young children. Additional long-awaited refinements [10, 11] are the representation of dynamic effects of transmission and immunity over time and variation in impacts depending on baseline endemicity, intervention coverage levels —based on statistical summaries of simulations [12–14] performed in the dynamic model OpenMalaria [15, 16].

This article presents and compares projections of the impact on under-five mortality of ITNs and effective malaria case management, performed in Spectrum-Malaria and in LiST, for a high-endemic and a low-endemic African setting. Differences and similarities are discussed with implications for interpreting modelling results for strategic planning. Options are discussed for updating and aligning both models and their input data and assumptions, and for operating both models in combination to cover the fullest set of interventions relevant for comprehensive program planning.

## Methods

Projections were made for the Democratic Republic of the Congo (DRC), as an example of a setting with stable high endemicity, and Zambia as an example of a setting with lower endemicity, over a 15-year horizon, and under-5 mortality impacts were compared between LiST and Spectrum-Malaria.

### Spectrum-Malaria projections

The Spectrum-Malaria model of 15th June 2016 (version 5.45 Beta 4) was used, detailed elsewhere [12, 14]. This includes statistical regression models that predict the proportional reduction in *Pf*PR, case incidence and malaria mortality, for a given change in intervention coverage, fitted to simulations of such intervention scenarios in the dynamic transmission model OpenMalaria [13]. Spectrum thus provides a simple, quick calculation tool for program planners that captures most of the dynamics of the full OpenMalaria research model. Interventions included were: ITNs, IRS, effective management of uncomplicated malaria cases (CMU) and of severe cases, and seasonal malaria chemoprophylaxis for children 3–59 months.

For simulations underlying Spectrum’s statistical estimates of ITN impacts, the effectiveness of ITNs (and IRS) in the OpenMalaria model had been calibrated [13] to fit the observed 2-year impacts on under-five mortality, case incidence and parasite infection prevalence from three ITN trials in Kenya and Ghana [17], that were also the basis for mortality effectiveness assumptions in LiST [9]. Effectiveness of management of uncomplicated malaria cases was modeled assuming a 100% cure rate for malaria patients complying with an effective antimalarial treatment, e.g. with an artemisinin-based combination therapy (ACT) [18].

Malaria mortality rates at 2014 and 2015 (and preceding years) were taken from the WHO’s official national estimates of November 2015 [1] for children under-5 versus older age groups. Baseline (2000–2015) data for the impact modifiers, seasonality in malaria transmission, and *Pf*PR in children 2–9 years, were taken from estimates by the Malaria Atlas Project [2, 19] —aggregated from 5 × 5 km^2^ pixel level estimates into first administrative level (Admin1) units (i.e. states or provinces). National-level impacts are computed by Spectrum-Malaria by aggregating burden and impact projections for each province in a country, thus taking account of (part of the) sub-national variation in impact determinants. The WHO’s national-level mortality estimates were distributed over Admin1 units by assuming a similar proportional distribution as estimated by the Malaria Atlas Project for malaria case incidence [2, 19].

For projections over 2016–2030, Spectrum applied proportional impacts predicted by regression models fitted to the average outcomes over years 1–3 after intervention scale-up from OpenMalaria simulations [13] over 2016–2021; for 2022–2030 Spectrum applied the proportional impacts from regression models fitted to average OpenMalaria simulation outcomes over years 8–10 after scale-up [13]. Spectrum applies these impact functions with a one-year time lag from intervention scale-up to start of impact, thus the projected burdens in 2016 reflected the effect of intervention coverage changes from 2014 to 2015, and onwards (Table 1).

**Table 1:** Key structural and methodological differences between LiST and Spectrum-Malaria

Aspect	LiST	Spectrum-Malaria
Health outcomes considered, that are impacted by malaria interventions	• Malaria-attributable, other-cause and all-cause mortality in children 0–4 years (separately for neonatal, 1–12 months and 13–59 months sub-groups) • Maternal deaths • Stillbirths	• Malaria-attributable mortality and case incidence, in 0–4 years, 5–14 years and 15+ years; • *Plasmodium falciparum* infection prevalence (*Pf*PR), in children 2–9 years
Interventions modelled	• Vector control (IRS and/or ITNs) • Case management, uncomplicated malaria cases • Intermittent Preventive Therapy for pregnant women	• ITNs • IRS • Case management, uncomplicated malaria cases (CMU) • Case management, severe malaria cases • Seasonal Malaria Chemoprophylaxis (SMC) in children 3–59 months
Determinants of impact of malaria intervention scale-up	Proportional reduction from baseline burden level, for a given proportional increase in intervention coverage – same within and across all countries	Proportional reduction from baseline burden level, varying with baseline endemicity (*Pf*PR), seasonality in malaria transmission, and baseline and scale-up coverage levels of ITNs, IRS, CMU and SMC, which act in interaction), calculated at province-level and then aggregated to national level [13]
Determinants of impact, for a given coverage increase	Fixed effectiveness value (for ITNs and CMU: from sources below), for all years and all countries [48]	• Baseline malaria endemicity (*Pf*PR in 2–9 years), • Seasonality in malaria transmission, • Baseline and scale-up coverage levels of other malaria interventions that have time-dynamic impacts (ITNs, IRS, SMC, and management of uncomplicated cases), and their variations between provinces, within and across countries [13].
Synergy or saturation across interventions?	No	Yes [13].
Saturation of incremental impacts at higher coverages?	No	Yes [13].
Time path of impact	No variation over time: impact is immediate from the year of scale-up; the post-intervention mortality level stays constant thereafter until coverage changes again	• Impact modelled with a 1-year lag after intervention scale-up; • Separate impact functions for short term (years 2–6 after scale-up) and long term (years 7–15 after scale-up) [12, 14].
Basis and source of coverage-impact relationship: ITNs	United Nationals Child Health Epidemiology Reference Group (CHERG), meta-analysis of randomized ITN trials [9]	Dynamic transmission model simulations for a wide range of sub-Sahara-Africa like scenarios, varying in endemicity, seasonality and baseline intervention coverages, performed in the OpenMalaria model – summarized in multi-variate statistical models [13]. OpenMalaria intervention effectiveness assumptions were in turn based on meta-analysis of randomized ITN trials [9], and review of observational treatment impact studies [18, 49]
Basis and source of coverage-impact relationship: case management	Meta-analyses of published observational studies and a previous Delphi estimate [21]	

Differences in data sources, intervention coverage definitions and country baseline parameter values are described in Tables 1 and 2

### LiST projections

LiST was used in the version ‘Spectrum 5.43 beta 1’ of May 2016. Outputs analyzed were the cause-specific deaths in children under five years (i.e. 0–59 months) of age. All-cause neonatal and post-neonatal mortality rates at 2014 and 2015 (and preceding years) were taken from estimates by the United Nations Inter-Agency Group for Child Mortality Estimation as of 2015 [20] (Table 2). The corresponding malaria mortality rates were derived by applying the proportion of post-neonatal under-5 deaths due to malaria (among eight other causes) from the WHO in the version as of October 2015 (http://www.who.int/healthinfo/global_burden_disease/estimates/en/index3.html) and [1].

**Table 2:** Parameters and input data used by LiST and Spectrum-Malaria in projections for DRC and Zambia

Parameter (2015, unless otherwise indicated)	DRC		Zambia		Data sources & definitions	
	Spectrum-Malaria	LiST	Spectrum-Malaria	LiST	Spectrum-Malaria	LiST
Population at malaria risk^a^	97.1%	91%	100%	98%	All-age population living where *Pf*PR among 2–9 years >0 [2, 19]	Women exposed to *P. falciparum* malaria [50]
Population 0–4 years (including children living not at malaria risk)	12,373,927	13,682,392	2,848,069	2,888,817	United Nations Population Division [51]	
Index for seasonality in malaria transmission	0.36	NA	1.64	NA	Coefficient of variation in EIR over a year, defined as the standard deviation divided by the year-average of monthly EIR [34]; country estimates from Malaria Atlas Project [2, 19]	
Prevalence of *P. falciparum* infection in children 2–9 years, average over 2000–2002	64%	NA	35%	NA	Malaria Atlas Project [19]	
Malaria deaths in children 0–59 months (% of all-cause deaths)	33,038	47,473 (16%)	2734	2723 (5.9%)	WHO (http://www.who.int/healthinfo/global_burden_disease/estimates/en/index3.html and [1])	WHO
All-cause under-5 deaths	NA	298,200	NA	45,916	NA	UN Inter-Agency Group for Child Mortality Estimation [20]
Malaria deaths in 5–14 years	3258	NA	2226	NA	WHO [1]; share of 5–14 versus 15+ years as for malaria cases [2, 19]	NA
Malaria deaths in 15+ years	2936	NA	2074	NA		
Malaria cases i.e. disease episodes in 0–4 years	8,231,156	NA	1,188,935	NA	WHO [1]; the share of 0–4 years in WHO’s all age estimate taken from Malaria Atlas Project [2, 19]	NA

*Abbreviations*: *EIR* Entomological Inoculation Rate, *LiST* Lives Saved Tool, *DRC* Democratic Republic of the Congo, *MAP* Malaria Atlas Project, *P. falciparum Plasmodium falciparum, NA* not available

^a^The population at risk of malaria does not influence impact calculations, but it is used in the OneHealth Tool costing as the ‘Population in Need’ (PIN) that would need to get various services like ITNs, IRS spraying, etc. (Equation for number of services: Target Population * PIN * Coverage)

Effectiveness of ITNs and/or IRS (with the coverage definitions detailed in Table 3), was estimated and implemented as children 1–59 months living in households protected by ITNs and/or IRS having a 55% lower risk of malaria-attributable death [9]. Treatment of *Plasmodium falciparum* malaria cases (without distinction between uncomplicated and severe cases) with artemisinin combination therapy (ACT) was assumed to reduce malaria mortality in children 1–23 months by 99% (range: 94–100%), and in children 24–59 months by 97% (range: 86–99%) [21].

**Table 3:** Intervention coverage at 2015 in DRC and Zambia, and two coverage-standardized country variants modelled

	Module	ITN coverage	IRS coverage	Malaria case management
DRC	Spectrum-Malaria	Usage: 55%	0.27%	6.6%
	LiST	ITN owning and/or IRS-sprayed: 70%		1.7%
Zambia	Spectrum-Malaria	Usage: 68.8%	35.6%	44.1%
	LiST	ITN owning and/or IRS-sprayed: 74.7%		18.4%
Coverage-standardized, DRC & Zambia^b^	Spectrum-Malaria	• 51% ITN usage at 2014 & 2015; • 70% ITN usage as target for 2016–2020; • IRS: 0.28% throughout 2014–2030.		• 10% at 2014 & 2015; • 40% as target for 2016–2020
	LiST	• 77% ITN owning and/or IRS sprayed at 2014 & 2015; • 98% as target for 2016–2020		• 10% at 2014 & 2015; • 40% as target for 2016–2020.
Data source & definition	Spectrum-Malaria	WHO/MAP 2015 [24], % of national population sleeping under an ITN (including areas with *Pf*PR = 0)*.*	WHO 2015 [1], % of national population protected (all ages, including areas with *Pf*PR = 0)	ACT treatment of RDT-positive (and RDT-negative) fevers in children 0–4 years [28], applied to uncomplicated malaria cases in all age groups
	LiST	Households owning ≥1 ITNs or protected by IRS as measured in the most recent national-representative household survey (usually MIS, MICS or DHS)^a^		Children 0–4 years with a fever treated within 48 h of fever onset with an artemisinin-containing compound i.e. ACT as measured in the most recent national-representative household survey (usually MIS, MICS or DHS)^a^

*Abbreviations*: *ACT* Artemisinin-based Combination Therapy, *DHS* Demographic and Health Survey, *DRC* Democratic Republic of the Congo, *ITN* Insecticide-treated mosquito net, *IRS* Indoor Residual Spraying, *LiST* Lives Saved Tool, *MAP* Malaria Atlas Project, *MICS* Multiple Indicator Cluster Survey, *MIS* Malaria Indicator Survey, *SSA* sub-Saharan Africa, *Swiss TPH* Swiss Institute of Tropical and Public Health, *PfPR Plasmodium falciparum* parasite prevalence rate

^a^LiST uses the measured values for years in which surveys occurred, and applies linear interpolation between measured points. For years after the last survey in a country, LiST assumes that coverage is constant

^b^Precise annual coverages for each intervention, scenario, and model are shown in Additional file 1

### Standardized intervention scale-up and scale-down scenarios

For the current model comparisons, baseline coverages and intervention scale-up targets for ITNs and CMU were standardized between LiST and Spectrum-Malaria (Table 3, row labeled ‘Coverage-standardized, DRC & Zambia’), and between the two countries, so as have a similar extent of scale-up from baseline to target level in both countries. Coverage values were set in the range of default coverage values assumed by the two models for the two countries, as explained in the following two paragraphs and with precise annual values shown in Additional file 1.

For vector control, LiST uses a combined coverage metric combining protection by ITNs and/or IRS, defined as the proportion of children under-5 who live in a household owning one or more ITNs, and/or in a house that has been sprayed with IRS within the past 12 months. Spectrum-Malaria uses as coverage metrics: for ITNs, the proportion of the population of all ages who slept under an ITN the last night; and for IRS, the proportion of the population of all ages who live in a house sprayed with IRS within the past 12 months. The standardized coverage assumptions we used for projections and comparisons were: for LiST, 70% ITN/IRS protection at 2014 and 2015 (close to the LiST default values of 70% for DRC and 74.7% for Zambia), increasing to 98% as the maximum target level (at 2016, or at 2020 with linear increase over 2016–2020); for Spectrum-Malaria, the standardized coverage assumption (judged most similar to standardized coverage in LiST) was 51% ITN usage at 2014 and 2015 (slightly below the Spectrum-Malaria default values of 55% for DRC and 68.8% for Zambia), increasing to 70% as maximum target level. We considered 51% and 70% usage to be similar to 77% and 98% of households owning one or more ITNs, based on ratios between these metrics in DRC, Zambia and other stable malaria-endemic African countries observed in national household surveys ([22–24] and Additional file 2), and therefore we consider these standardized coverage values optimal for the purpose of comparing impact projections despite the different coverage metrics in the two models. In Spectrum-Malaria, IRS coverage was kept constant throughout 2014–2030, so that IRS coverage did not have any projected impact.

For malaria case management, Spectrum-Malaria distinguishes between uncomplicated cases and severe cases, but LiST does not. We compared management of uncomplicated cases from Spectrum-Malaria with overall case management from LIST. For these respective interventions, both models use as input the coverage of child fevers treated with an ACT, as observed in national household surveys: most often, Demographic and Health Surveys (DHS) [25], Multiple Indicator Cluster Surveys (MICS) [26] and Malaria Indicator Surveys (MIS) [27]. LiST inputs new survey data points individually and continually as these survey data are released, while Spectrum-Malaria pre-loaded a multi-country dataset of survey-based coverage estimates for 2005, 2010 and 2015, in which survey data were adjusted and extrapolated in a standardized way to years and countries without surveys [28]. In Spectrum-Malaria for most African countries including DRC and Zambia, less than 8% of cases are severe [12, 14], so the difference in denominators (uncomplicated cases versus all malaria cases) should not invalidate the comparison of their impacts. For the current projections, coverage in both DRC and Zambia was set at 10% for 2014 and 2015, increasing to 40% as maximum target level (again reached either in 2016, or in alternative scenarios by 2020 with linear scale-up over 2016–2020).

Using these target coverage levels for ITNs and CMU, we modelled and compared the following 6 scale-up scenarios:

- Scaling-up ITN to the stated target coverage;
- Scaling-up CMU to the stated target coverage;
- Scaling-up ITNs and CMU to the stated respective target coverages, in parallel; with for each of these 3 intervention targets: either scale-up at once realized in 2016 and maintained throughout 2030, or a linear scale-up starting in 2016 with the target coverage level reached by 2020 and then maintained until 2030.

In addition, to show the coverage-to-impact relationship over a wide coverage range, including the effect of scaling-down coverage to below the 2014–2015 baseline level, several scale-down scenarios were modelled (details in Additional file 1), with each coverage change implemented at once in 2016 and sustained until 2030.

### Health outcomes and time horizons evaluated

Impacts were compared as proportional reductions in under-5 mortality numbers, so that some small differences in baseline mortality rates between the two models (Table 2) did not affect the comparison.

We evaluated mortality impacts over 2016–2030, the horizon considered in Spectrum-Malaria (while LiST does not specify a specific horizon of validity). Some comparisons focused on either 2020 or 2025, as indicative of shorter-term impacts versus longer-term impacts, respectively, being modelled through distinct statistical impact functions in Spectrum-Malaria [13].

## Results

### Scaling-up ITN and CMU from 2015 coverage levels

**Fig. 1:**
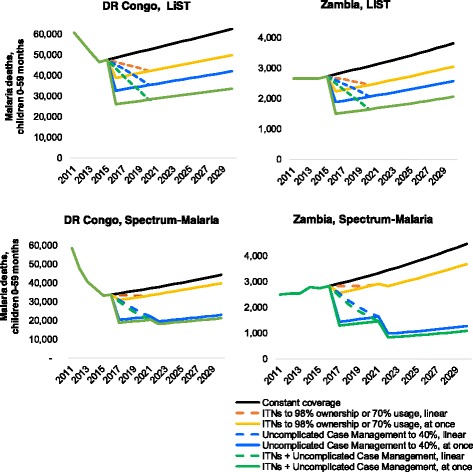
Impacts over time, for ‘Coverage-standardized’ country variants of DRC and Zambia. Constant coverage as defined in Table 3 for year 2015, Coverage-standardized variants of DRC and Zambia. For ITN + CMU combined (green lines), target coverages (for years 2016 and onward) are 98% ITN ownership in LiST or 70% ITN usage in Spectrum-Malaria, and 40% CMU coverage

In both LiST and Spectrum-Malaria, increasing CMU coverage from 10% to 40% has larger impact than increasing ITN coverage from 77% ownership or 51% usage to 98% ownership or 70% usage (Figure 1).

**Fig. 2:**
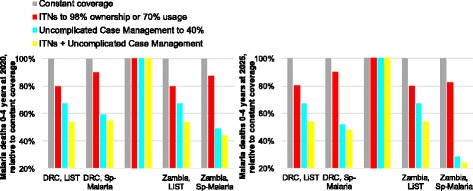
Deaths in 0–4 year-olds, relative to constant-coverage scenario, (left) 2020 and (right) 2025. Constant coverage as defined in Table 3 for year 2015, Coverage-standardized variants of DRC and Zambia. For ITN + CMU combined (yellow bars), target coverages (for years 2016 and onward) are 98% ITN ownership in LiST or 70% ITN usage in Spectrum-Malaria, and 40% CMU coverage

In LiST, intervention scale-up reduces mortality to a new post-intervention level within the same year as the increased intervention coverage; this new mortality rate is maintained over next years and annual numbers of deaths reflect this rate multiplied by the (growing) population size. In Spectrum-Malaria, intervention scale-up starting in 2016 results in a reduced mortality rate over 2017–2021, with a further reduced mortality rate over 2022–2030 reflecting the long-term transmission dynamic effect. For ITNs, proportional under-5 mortality reductions following scale-up are somewhat larger in LiST than in Spectrum-Malaria, for both DRC and Zambia in the short-term over 2016–2021 (Fig. 2, left). Over 2022–2030, proportional ITN impacts are similar between both models for Zambia, but still larger in LiST for DRC (Fig. 2, right).

For CMU, in contrast, Spectrum-Malaria predicts larger proportional mortality reductions than LiST, for both countries, across 2016–2030 and throughout the coverage range of 0–100%, but especially so for lower-endemic Zambia and for the longer (2022–2030) time horizon.

For both interventions, LiST projects identical proportional impacts for a given coverage increase in DRC and Zambia (Fig. 2 & Table 4), and across all other countries (not shown). Spectrum-Malaria, in contrast, projects larger proportional burden reductions for the lower-endemic Zambia, compared to the higher-endemic DRC.

**Table 4:** Relative under-5 malaria mortality levels after simultaneous scale-up of CMU and ITNs

Scale-up scenario	LiST		Spectrum-Malaria	
	DRC	Zambia	DRC	Zambia
ITN 77% ownership or 51% usage; Uncomplicated Case Management 10% (i.e. constant coverage scenario of Figs. 1, 2 and 3)	100%	100%	100%	100%
ITN 98% ownership or 70% usage (single intervention)	80%	80%	90%	88%
Uncomplicated Case Management 40% (single intervention)	67%	67%	59%	49%
ITN 98% ownership or 70% usage & Uncomplicated Case Management 40% (two interventions concurrently)	53.8%	53.8%	54.7%	43.8%
Product of Relative mortality levels after ITN scale-up (to 98% ownership or 70% usage) alone, and after scale-up of Uncomplicated Case Management (to 40%) alone	53.9%	53.8%	53.2%	42.8%

The results percentages reflect the mortality level at 2020 in the scale-up scenario, relative to the mortality level at 2020 in the scenario with coverages held constant at 2015 levels. Each coverage scale-up was implemented as an immediate coverage increased from 2015 to 2016, and maintained over 2016–2030. ITN ownership indicates coverage level projected in LiST; ITN usage indicates coverage level projected in Spectrum-Malaria

### Scaling-down ITN and CMU from 2015 coverage levels

**Fig. 3:**
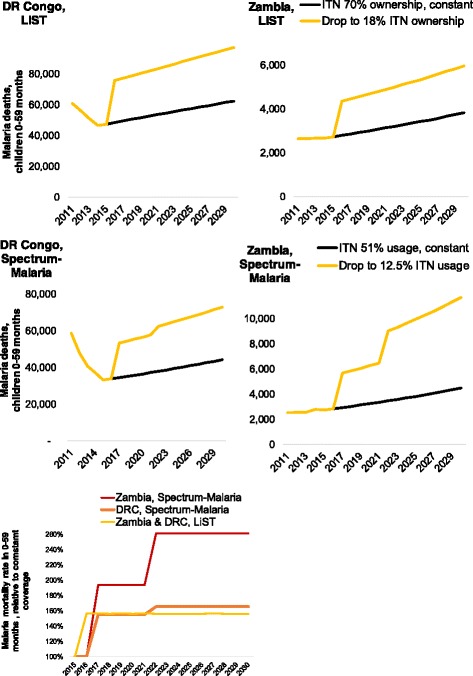
Impacts of ITN scale-down from 2016, for ‘Coverage-standardized’ variants of (left) DRC and (right) Zambia. (top) LiST, malaria deaths and (middle) Spectrum-Malaria, malaria deaths; (bottom): LiST and Spectrum-Malaria, Malaria mortality rate relative to the constant-coverage scenario. Scale-down scenarios shown entailed a drop from 51% to 12.5% in ITN utilization in Spectrum-Malaria, or from 77% to 18% ITN ownership and/or IRS sprayed in LiST

For scenarios decreasing ITN coverage to below the 2015 actual coverages (which were already high in both DRC and Zambia, and in our coverage-standardized variants of these countries), Spectrum-Malaria predicts a similar mortality increase as LiST for DRC over the first five years after ITN scale-down (represented as a drop from 51% to 12.5% utilization, or from 77% to 18% ownership), followed by a further mortality rise compared to LiST from the 7th year following scale-down (Fig. 3). For Zambia, in the short-term (over 2016–2021) and especially the longer-term (over 2022–2030), the projected mortality rise due to ITN coverage decrease is larger in Spectrum-Malaria than in LiST.

### Non-linearity in the coverage-impact relationship

**Fig. 4:**
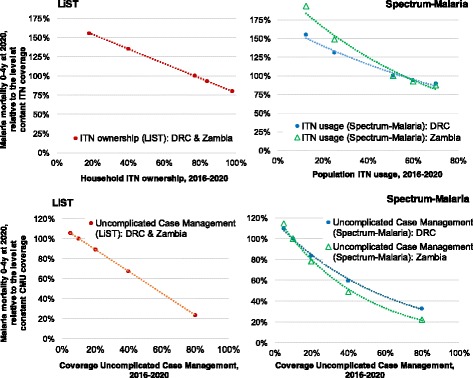
Non-linearities in mortality impacts of ITNs and CMU, following scale-up or scale-down at 2016**.** The y-axes express the mortality level at 2020, in the scale-up or scale-down scenario, relative to the mortality level projected at 2020 under ‘constant’ coverages as shown in Figs. 1, 2 and 3: CMU coverage is kept constant at 10%, ITN household ownership at 77% (in LIST) and ITN usage at 51% (in Spectrum-Malaria); the mortality level at these coverage levels are each displayed as 100%, so as to allow displaying both countries within the same chart despite their differing malaria mortality levels

We compared mortality impacts for a given coverage increase or decrease, over the full 0–100% range of possible coverages and between the countries, as comparative proportional mortality reductions relative to the mortality level under a constant-coverage scenario, at the year 2020. In LiST, the effect of a given coverage increase is calculated as a simple multiplication of (reduced) relative risks that are linear with the coverage increase, identically for all countries, as shown in Fig. 4, with identical lines for DRC and Zambia. Spectrum-Malaria, in contrast, shows larger proportional impacts of both ITN and CMU for the lower-endemic Zambia (steeper curves in Figs. 4b and d) than for higher-endemic DRC. Spectrum-Malaria furthermore shows an important saturation effect apparent at coverage levels of around 50% or higher, for both interventions: adding 20 percentage points ITN or CMU coverage from a moderately high baseline level of e.g. 50% coverage does not produce as large a mortality reduction as adding 20 percentage point coverage from a low baseline level of e.g. 10%.

### Interaction between ITNs and CMU

A non-linearity that is captured in Spectrum-Malaria but not in LiST is interaction between interventions, for example between ITNs and CMU. In LiST, the proportional impacts of concurrent ITN/IRS and CMU scale-up equate the product of the proportional impacts of the two interventions when implemented each in isolation (Table 4). In Spectrum-Malaria, in contrast, for both DRC and Zambia, concurrent scale-up to high coverage levels for ITNs and CMU simultaneously entails a slight saturation in the overall impact, with the overall proportional reduction slightly less than the product of the corresponding relative reductions achieved by the two interventions when each applied alone.

## Discussion

The presented projections of impacts of ITNs and CMU on malaria mortality from two models show how Spectrum-Malaria usefully corroborates and complements LiST, in projecting impacts of key malaria interventions including their variation over time with some dynamic transmission effects materializing from six years after scale-up, variation by baseline endemicity and as a function of baseline coverage levels, and dynamic interactions between interventions. Overall, Spectrum-Malaria broadly supports the magnitude of mortality impacts of ITNs and CMU projected by LiST [3] and several retrospective impact evaluations [2, 29, 30] for *falciparum*-endemic African settings. The similar proportional mortality reductions over the first two years following scale-up of ITNs from near-zero baselines to moderately higher coverages are as expected, since both models were validated on cluster-randomized ITN trials in moderate-to-high-endemic settings with a 2-year duration. The ITN-related mortality reductions projected are also in line with magnitudes of mortality decline associated with the increased malaria donor funding over the 2000s, which had been allocated primarily to ITN distribution programs and improved case management [31, 32].

In comparison, a recent assessment of impacts expected over 2015–2030 from scaling-up malaria control according to the WHO’s Global Technical Strategy, using the Imperial College London malaria transmission model, projected a possible reduction in malaria mortality rates of 40% (across all ages) from 2010 to 2030 across 80 countries with sustained stable malaria transmission in 2010 [33]. This would seem to be a smaller impact than projected by LiST and especially Spectrum-Malaria for the combined scale-up of ITN and CMU in DRC and Zambia (Fig. 2). Strict quantitative comparison should rather be pursued country by country and age group by age group, for standardized intervention packages and coverage increments, as recently done with OpenMalaria, the Imperial College London model and two other dynamic models for the child health impacts of RTS,S vaccination [34]. That comparison illustrated that uncertainties about malaria pathogenesis as a function of acquired immunity, and the resulting age distribution in malaria burdens, especially in low-transmission settings, leave a considerable margin of uncertainty about the magnitude of (proportional and absolute) health impacts of malaria control, and about effectiveness rankings among interventions. In this context, the projection results that we presented for DRC and Zambia in fact seem quite consistent between LiST and Spectrum-Malaria.

Importantly, the Spectrum-Malaria projections suggest that the gains expected from ongoing ITN scale-up toward universal coverage —as targeted now by the WHO’s Global Technical Strategy for Malaria 2016–2030 — may be somewhat smaller than predicted by LiST, while the potential impact of CMU may be larger. In drawing national strategic plans for malaria control, program planners should, however, consider the modelled ranking of impacts alongside mapping of the cascades of services, activities and inputs needed to achieve a given coverage level and increase —which for ITNs (through vertical distribution campaigns) may be easier than for CMU (which typically requires a broader health systems strengthening). Tellingly, of the 663 million malaria cases averted in sub-Saharan Africa due to malaria control interventions over 2000–2015, it was estimated that 69% were averted due to use of ITNs, and only 21% due to ACT-based treatment, and 10% due to IRS [2]. Spectrum-Malaria can support comprehensive impact and cost-effectiveness evaluation through its One Health Tool module, which links with impacts projected through LiST and the Spectrum-Malaria module.

The structural differences between Spectrum-Malaria and LiST (Table 1) underlie several important differences in impact results, besides different (generally larger) long-term impacts compared to short-term population level impacts. First, Spectrum-Malaria projects proportionally larger burden reductions for settings with lower baseline burdens. This was evident in the current projections as larger proportional mortality reductions for Zambia compared to DRC, and it applies to other settings and other malaria-related health outcomes as well, being a function of statistical relationships driving impact projection in Spectrum-Malaria, which include a negative coefficient for *Pf*PR, so that settings with higher *Pf*PR will systematically have lesser proportional burden reductions for any given coverage increase [13]. Of note, while this pattern was illustrated here as a difference between Zambia and DRC, the endemicity effect is modelled in Spectrum-Malaria at the sub-national level (of Admin1 units, i.e. first sub-national level), so that within Zambia, within DRC or within any other country, the proportional health impacts are larger in lower-endemic provinces or states than in those with higher endemicity. This modelled pattern is consistent with observations from ITN trials [17] and with models of the dynamics induced by various malaria interventions [34–37]. In contrast, absolute health gains —in terms of cases and deaths averted for a given coverage increase— are generally larger for higher-endemic settings (e.g. DRC more than Zambia), due to their larger baseline burden compared to lower-endemic settings.

Second, more influentially, in Spectrum-Malaria the incremental impacts for a given coverage percentage increase saturate at higher coverages. LiST, in contrast, by default calculates impacts fully linearly with coverage increases throughout the 0–100% coverage range. And a special option in LiST called ‘transmission herd effect’ allows users to even amplify impacts of ITN and/or IRS protection by specifying proportions of children who are themselves uncovered by ITN and/or IRS to still be protected from malaria – but there are no default assumptions for this effect, and the herd effect option cannot model negative (saturating) effects of saturating ITN or IRS coverage.

Third, in Spectrum-Malaria, the combined impact of simultaneous scale-up of multiple interventions does not necessarily equate the product of the individual interventions’ proportional impacts (as in LiST), but this can be less due to saturation effects, as shown for concurrent ITN and CMU scale-up in DRC and Zambia (Figure 3). In other settings, notably higher-endemic settings with lower baseline and target coverages (not shown), Spectrum-Malaria projects synergy between interventions, whereby combinations of interventions reduce transmission and endemicity more strongly, and the incremental effect of additional interventions then amplifies the overall effect beyond the contributions of single interventions [13]. These saturation and synergic effects reflect the dynamics of malaria control, as simulated in OpenMalaria [38] and emulated through Spectrum’s statistical impact functions [13].

Fourth (less relevant to the child health perspective and comparison with LiST, but important for overall infectious disease and health sector planning) Spectrum-Malaria projects important impacts of malaria control on morbidity and mortality in children above five years and adults. These older age groups have much lower shares in malaria morbidity and mortality burdens, which malaria intervention coverage scale-up would reduce by near-similar proportions as shown here for children under-5 —but with some rebounds in longer-term reflecting declining acquired immunity in cohorts of people benefiting from enhanced malaria protection [13]. As a result, Spectrum-Malaria, like the dynamic transmission models that informed it, predicts that enhanced malaria control will progressively shift malaria burden to older ages and result in a more homogeneous distribution of malaria over age [39] (as already seen in lower-endemic settings outside of Africa), heightening the importance of including over-fives in impact evaluation and strategic program planning.

Finally, LiST remains unique in examining the impact of malaria interventions within the context of all causes of child mortality. Impacts of malaria interventions on absolute numbers of deaths are influenced by interventions that affect those other causes, as they affect the number of children exposed to malaria mortality. This enables comparisons of malaria interventions with other child survival interventions and focuses attention on opportunities currently being missed for reducing child mortality, by targeting the major causes of child deaths for which effective interventions exist but are not yet implemented to scale.

### Limitations

Limitations of the Spectrum-Malaria and LiST tools, that influence the projection results compared here, have been discussed elsewhere [10–12]. These include that country under-5 malaria mortality data used for the 2015 baseline were themselves model-based and subject to uncertainty and annual re-estimation [1, 40–42]. Further in terms of data inputs, coverage definitions and the assumed baseline and target levels were not necessarily comparable between both models. For ITNs, the relationship between household ownership of ≥1 ITNs (used in LiST) and usage (used by Spectrum-Malaria) is well-characterized by recent multi-country analyses, but this may change as coverage increases and over-allocation to households with already enough ITNs, at the expense of less accessible households who still lack ITNs, increases.

For CMU, both models used coverage estimates for children under-5 years, but LiST assumed no treatment in older age groups, and considered only treatments within 48 h of onset of fever, while the CMU coverages used by Spectrum-Malaria ignored timeliness of treatment but focused on fevers with RDT-confirmed parasitemia [28]. It is not clear how the two sets of coverage estimates relate, and this uncertainty compounds large uncertainty in effectiveness of CMU.

Malaria treatments in older age groups modeled in Spectrum-Malaria (at the same coverage as for children under-5) but not in LiST, including their dynamic transmission-reducing effects over time, explains why Spectrum-Malaria projected larger long-term impacts of CMU —especially in low-endemic Zambia where high coverage of CMU might, in some of the lower-endemic areas within the country, feasibly drive malaria transmission down to pre-elimination levels. It is hard to say which model is more realistic here, lacking cluster-randomized trials of population-level CMU impacts. The range of impacts between the two models may well capture the real typical magnitude —as well as the uncertainty or variation— in CMU impacts. A more comprehensive, refined comparison between both models of health impacts and cost-effectiveness of CMU should furthermore take into account the required volumes of treatments (across all ages) and other program inputs involved in the respective CMU scale-ups projected. Both models will likely benefit from aligning CMU coverage baseline data, building on progressively improving estimates of effective CMU coverage for parasitologically confirmed malaria to replace current coverage estimates based on child fevers from any cause, that also take into account antimalarial drug availability, quality, patient adherence and compliance, timeliness, caretakers’ recall of treatment histories in household surveys [43], and sub-national geographical variation in these determinants of CMU coverage [2, 28, 44]. Such forthcoming refined CMU coverage estimates will also be used for ecological, statistical assessments of historic CMU impacts over space and time in Africa (as recently done for ITNs, IRS and ACT access [2]), which may provide an opportunity to triangulate and validate CMU effectiveness assumptions.

For ITNs, while effectiveness has been fairly precisely estimated for scale-up as occurred over the past decade, and both LiST and Spectrum-Malaria fitted to that, the incremental impacts of ongoing further ITN scale-up may saturate with increasing coverage, which was captured in Spectrum-Malaria but not in LiST. Finally, ITN and IRS impacts will diminish as insecticide resistance develops and spreads. Insecticide resistance was not captured in either model, but should be added as an impact modifier when good-quality standardized data on WHO-recommended resistance indicators become available at country and sub-national resolution [45]. Similarly, CMU effectiveness may be lower in settings with parasitological resistance against the locally used antimalarial drugs [1], an effect modifier not yet captured in either model’s impact assumptions.

### Implications for program planning

Going forward, to optimally use LiST and Spectrum-Malaria for program and health sector planning, we recommend that program planners use both models in complement. Spectrum-Malaria probably gives the best comprehensive impact projection for ITNs, IRS and CMU —including dynamic transmission effects as they evolve over time and over successive phases of malaria control, and vary with baseline epidemic conditions of each settings. Spectrum-Malaria adds to the arsenal of malaria interventions projected with seasonal malaria chemoprophylaxis (recommended in countries and areas with strongly seasonal malaria, mainly in Western and Sahelian Africa) and with improved management of severe malaria cases. LiST, but not Spectrum-Malaria, models the impact of Intermittent Preventive anti-malarial Therapy for pregnant women (IPTp), which benefits women receiving this intervention, and their infants directly. While the population-level impacts of IPTp (which is not known to produce onward dynamic transmission effects at population level) are small compared to transmission-reducing interventions that are the focus of Spectrum-Malaria, IPTp is an essential component of maternal, neonatal and child health care packages, that requires evaluation in the LiST context considering maternal, neonatal and child health outcomes specifically. And only LiST models malaria mortality in the context of other-cause under-five mortality, allowing validation against field program data that typically measure and monitoring all-cause under-five mortality without ascertainment of causes [46]. While Spectrum-Malaria primarily focuses on modeling dynamic interactions among malaria interventions, LiST provides a framework that more easily allows evaluations across a wider set of causes of child deaths with their possible interactions. For example, recent evidence suggests a link between sub-optimal breastfeeding and increased risk of malaria mortality in children under the age of six [47]. Such effects can be captured using the LiST approach, but would not be straightforward to account for in Spectrum-Malaria.

To facilitate the complementary use of LiST and Spectrum-Malaria, modelers should align the baseline country data included in both models, i.e. malaria mortality rates, populations living at risk of *Plasmodium falciparum* transmission, and recent coverage levels and trends of ITNs, IRS and CMU. To do cost comparison with both models jointly, also their assumed proportions of population living at risk of malaria should be aligned, since these determine the population in need of malaria interventions. Comparison and alignment may be further facilitated if Spectrum-Malaria would compute and display health impacts for pregnant women, as a distinct sub-group of adults. A future way to incorporate in LiST the non-linearity in coverage-impact relationships, and saturation and synergy across malaria interventions as illustrated by Spectrum-Malaria, would be for LiST to extend the herd effect c.q. transmission interruption option, that LiST currently allows for modelling of ITNs/IRS but not yet for malaria treatment.

## Conclusion

In conclusion, Spectrum-Malaria aligns with LiST in the proportional under-5 mortality reduction following ITN scale-up from low (0–20%) to current/high ITN coverage, as expected given that both models were fitted to the results of the same ITN trials. However, for scale-up from current intermediate coverage levels to universal coverage, Spectrum-Malaria suggests that important country variations and saturation effects in impacts are expected that are not evident from LiST. For CMU, Spectrum-Malaria predicts somewhat larger proportional impacts than LiST, which may reflect real long-term dynamic effects, although the precise impact of CMU remains uncertain lacking a controlled-trial-based gold standard. Strategic planners in malaria-endemic African countries can use the two models in a complementary way, within the Spectrum platform, to inform various malaria and health sector strategic planning purposes, including those focusing on population level health beyond child mortality.

## Additional files

Additional file 1: Coverage values assumed in LiST and Spectrum-Malaria impact projections, for Coverage-standardized variants of DR Congo and Zambia (XLSX 11 kb)

Additional file 2: Relationship between household possession of 1 or more ITNs and population (all ages) sleeping under an ITN, and coverage values used in LiST and Spectrum-Malaria projections for the Democratic Republic of the Congo (DRC) and Zambia. (XLSX 18 kb)

## Abbreviations

ACT: Artemisinin-based combination therapy
Admin1: First order administrative level geographical units (i.e. states or provinces)
CMU: Effective management o uncomplicated malaria cases
DHS: Demographic and Health Survey
DRC: Democratic Republic of the Congo
EIR: Entomological inoculation rate
IRS: Indoor residual spraying
ITN: Insecticide treated mosquito net
IPTp: Intermittent preventive antimalarial therapy for pregnant women
LiST: Lives Saved Tool
MAP: Malaria Atlas Project
MICS: Multiple Indicator Cluster Survey
MIS: Malaria Indicator Survey
*Pf*PR: *Plasmodium falciparum* parasite prevalence
RDT: Rapid Diagnostic Test of malaria parasitology
SSA: Sub-Saharan Africa
Swiss TPH: Swiss Tropical and Public Health Institute
WHO: World Health Organization.
